# Sinonasal Squamous Cell Carcinoma Survival Outcomes Following Induction Chemotherapy vs Standard of Care Therapy

**DOI:** 10.1177/01945998221083097

**Published:** 2022-03-08

**Authors:** Alexander T. Murr, Nicholas R. Lenze, Jared M. Weiss, Juneko E. Grilley-Olson, Shetal A. Patel, Colette Shen, Bhishamjit S. Chera, Adam M. Zanation, Brian D. Thorp, Siddharth H. Sheth

**Affiliations:** 1 Department of Otolaryngology–Head and Neck Surgery, The University of North Carolina at Chapel Hill, Chapel Hill, North Carolina, USA; 2 Division of Medical Oncology, Department of Medicine, The University of North Carolina at Chapel Hill, Chapel Hill, North Carolina, USA; 3 Department of Radiation Oncology, The University of North Carolina at Chapel Hill, Chapel Hill, North Carolina, USA

**Keywords:** sinonasal cancer, squamous cell carcinoma, induction chemotherapy, survival outcomes

## Abstract

**Objective:**

To compare oncologic outcomes in sinonasal squamous cell carcinoma (SNSCC) treated with standard of care (SOC) definitive therapy, consisting of surgery or chemoradiotherapy, vs induction therapy followed by definitive therapy.

**Study Design:**

Retrospective review.

**Setting:**

Academic tertiary care hospital.

**Methods:**

The medical records of patients with biopsy-proven SNSCC treated between 2000 and 2020 were reviewed for demographics, tumor characteristics, staging, treatment details, and oncologic outcomes. Patients were matched 1-to-1 by age, sex, and cancer stage according to treatment received. Time-to-event analyses were conducted.

**Results:**

The analysis included 26 patients with locally advanced SNSCC who received either induction therapy (n = 13) or SOC (n = 13). Baseline demographics, Charlson Comorbidity Index, and median follow-up time were well balanced. Weekly cetuximab, carboplatin, and paclitaxel were the most common induction regimen utilized. Tolerance and safety to induction were excellent. Objective responses were observed in 11 of 13 patients receiving induction. No difference in disease-free survival was found between the induction and SOC groups at 1 or 3 years. However, when compared with SOC, induction therapy resulted in significant improvement in overall survival at 2 years (100% vs 65.3%, *P* = .043) and 3 years (100% vs 48.4%, *P* = .016) following completion of definitive therapy. Two patients in the SOC group developed metastatic disease, as compared with none in the induction group.

**Conclusions:**

Induction therapy was safe and effective. When compared with SOC, induction therapy improved 3-year overall survival.

Sinonasal squamous cell carcinomas (SNSCCs) are rare malignancies that represent about 3% of all head and neck cancers.^1,2^ Definitive therapy with either primary surgery or concurrent chemoradiation therapy is the standard of care (SOC).^3-7^ Despite improvements in both these modalities, the treatment of advanced locoregional disease results in high morbidity and a 5-year overall survival (OS) of 30%.^2,4,8^

Sinonasal malignancies frequently develop in close proximity to important anatomic structures, posing significant challenges for treatment. For example, tumor involvement of the orbit necessitates orbital exenteration to ensure optimal surgical outcomes (R0 resection). Without randomized clinical trials investigating the efficacy of induction chemotherapy (IC) in SNSCC treatment, data supporting the use of IC in SNSCC are supported by retrospective studies and prospective studies involving mucosal head and neck cancers.^9-15^ Despite the limited data, IC can be utilized as a strategy to promote organ preservation.^16-18^ A review of 46 patients with untreated SNSCC found that a partial or complete tumor response to IC was associated with improved survival rates and the possibility for organ preservation.^19^ Furthermore, the use of IC may reduce the risk of metastasis from locoregionally advanced disease,^20^ with tumor response to IC possibly aiding in prognostication.^18,19,21-23^ Additionally, a meta-analysis of 7 studies and 423 patients^24^ found that stable or progressive disease following IC was associated with a poorer response to CRT.

We report our institutional experience with IC in the treatment of SNSCC. Using a 1:1 matched analysis, we compare oncologic outcomes in SNSCC treated with SOC definitive therapy vs IC followed by definitive therapy.

## Materials and Methods

An Institutional Review Board–approved retrospective review was conducted with a prospective institutional database of patients treated for sinonasal and skull base tumors at the University of North Carolina. Between 2000 and 2020, 92 patients with biopsy-proven SNSCC were treated at our institution. Demographic information was reviewed, including comorbidities, tumor characteristics, staging, treatment details, and oncologic outcomes. Of the 92 patients, 13 were treated with IC. The IC cohort subsequently underwent 1:1 matching for TNM stage (American Joint Committee on Cancer), age, sex, and race with the 72 patients who received the SOC (control group) within the same period.

All patients had locoregionally advanced SNSCC (T3-T4) and were treated with curative intent. Of note, p16 status was not consistently available with tumor pathology and was thus not available for analysis. Definitive therapies permitted were either primary surgery followed by adjuvant therapy (if indicated) or concurrent chemoradiotherapy. IC response was assessed through radiographic comparison of images prior to and following termination of IC. Tumor response was defined according to RECIST (Response Evaluation Criteria in Solid Tumors). Time-to-event analyses were conducted with main outcome measures, including OS, disease-free survival (DFS), and response to IC. To account for the effect of coexisting diseases on mortality, we used the Charlson Comorbidity Index (CCI), which predicts 10-year survival based on 19 comorbid factors. Higher scores correlate with an increase in mortality.^25^ The CCI has been validated when combined with age as a covariate^26^ and can independently predict survival following surgery for head and neck cancer.^27^ Additional post hoc analyses were performed with a median CCI score to assess the distribution of comorbidity burden: scores ≥4 were defined as low and >4 as high.

### Statistical Analysis

Descriptive statistics were used to compare baseline characteristics in the control and induction groups. Bivariate testing methods consisted of a 2-sided *t* test, chi-square test, and Fisher exact test. Kaplan-Meier curves were used to compare OS and DFS in the induction vs control cohorts. Subset analyses based on Kaplan-Meier curves assessed the impact of comorbid conditions on OS and DFS by comparing high and low CCI scores. The log-rank test was used to compare survival curves. Statistical significance was set at *P* < .05 of all analyses. Stata 16.0 (StataCorp LP) was used for all analyses.

## Results

### Patient Characteristics

Twenty-six patients were included and equally distributed in 2 groups: IC (n = 13) and control (n = 13). Of the 26 patients, 24 received treatment for their primary disease, while 2 were treated for recurrent disease (1 patient in each group). Baseline patient characteristics, including sex and cancer stage, were well matched in both treatment groups, with a majority having stage IVA or IVB disease. Mean (SD) age at the time of surgery was similar: 61 (11.7) years for the control group and 55 (10.2) for the IC group. The mean CCI scores were 4.62 (1.66) for the IC group and 3.77 (1.48) for the control group (*P* = .183), suggesting that comorbidity burden was not a major confounding variable (**Table 1**).

**Table 1.** Patient Baseline Characteristics.^a^

	Control (n = 13)	Induction (n = 13)
Age, y	61 (11.7)	55 (10.2)
Sex		
Male	7	7
Female	6	6
Race		
White or Caucasian	11	9
White Hispanic or Latino	1	0
Black or African American	1	3
Asian or Pacific Islander	0	1
Disease status		
Primary	12	12
Recurrent	1	1
TNM		
T3N0M0	0	1
T4aN0M0	10	9
T4bN0M0	1	2
T4bN2M0	2	1
AJCC stage, 7th ed		
III	0	1
IVA	10	9
IVB	3	3
CCI score^b^	4.62 (1.66)	3.77 (1.48)
Smoking status		
Active smokers	9	8
Former smokers	0	1
Never smoker	4	4
Pack-years: all patients	18.5	14
Pack-years: only smokers	27.75	20.3

Abbreviations: AJCC, American Joint Committee on Cancer; CCI, Charlson Comorbidity Index.

a Values are presented as mean (SD) or No.

b *P* = .183.

The most common reason for treatment with induction therapy was either organ preservation or improving the chance of obtaining negative surgical margins. Ten patients received multiagent systemic therapy consisting of carboplatin and paclitaxel with cetuximab (n = 8) or without (n = 2). Three patients, all treated before 2010, received concurrent cisplatin (weekly or bolus) with radiation therapy as induction therapy prior to definitive surgery. Tolerance to induction therapy was excellent. Only 3 patients experienced grade 3 toxicities secondary to IC treatment: neutropenia, type 1 hypersensitivity reaction, and acute kidney injury resulting in dose reduction (Supplemental Table S1, available online). No grade 4 or 5 toxicities were observed. The objective response rate was 84.6%, with 1 complete response, 10 partial responses, 1 stable disease, and 1 progressive disease. Within the control group, 10 patients were treated with surgery while the remaining 3 patients received chemoradiotherapy for definitive therapy (Supplemental Table S2).

### Survival Outcomes

**Figure 1.**
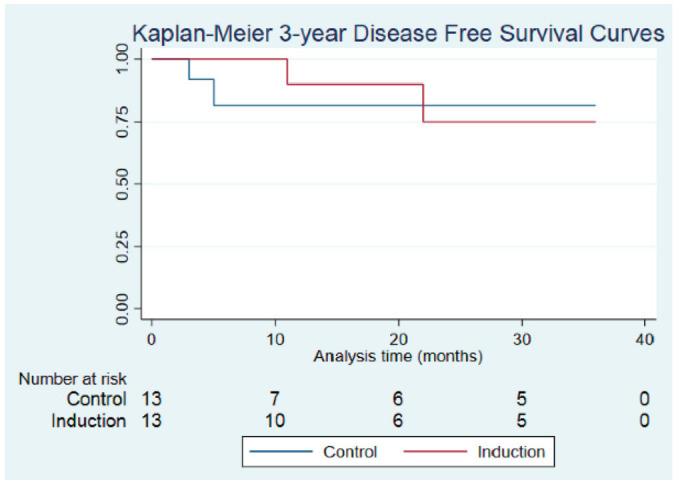
Comparison of disease-free survival in the control and induction chemotherapy (IC) groups: 1 year (90.0%, IC; 81.5%, control; *P* = .428), 2 years (75.0%, IC; 81.5%, control; *P* = .857), and 3 years (75.0%, IC; 81.5%, control; *P* = .867).

**Figure 2.**
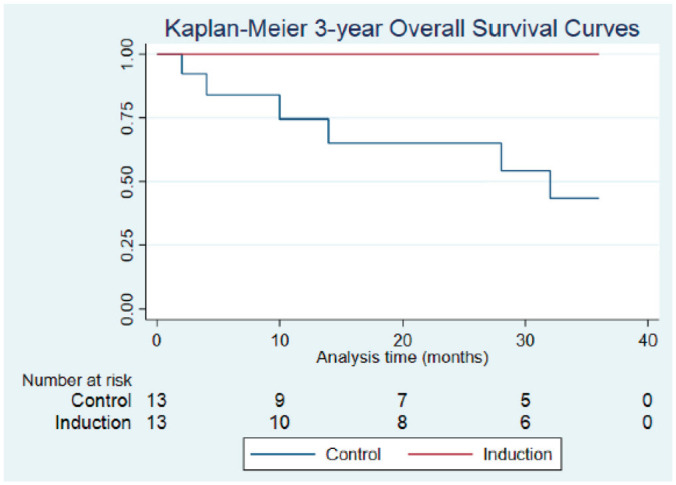
Comparison of overall survival in the control and induction chemotherapy (IC) groups: 1 year (100%, IC; 76.2%, control; *P* = .088), 2 years (100%, IC; 65.3%, control; *P* = .043), and 3 years (100%, IC; 48.4%, control; *P* = .016).

Mean (SD) follow-up was 46.4 (48.0) months following definitive therapy, and median follow-up was 21 (49.2) and 26 (48.6) months for the IC and SOC groups, respectively. Treatment with IC did not improve DFS as compared with SOC at 1, 2, or 3 years (**Figure 1**); however, at the 2- and 3-year intervals, OS was significantly higher in patients treated with IC vs SOC. At 1 year, OS was 100% and 76.2% (95% CI, 42.7%-91.7%) for the IC and control groups (*P* = .088), respectively. At 2 years, OS was 100% and 65.3% (95% CI, 31.4%-85.5%) for the IC and control groups (*P* = .043). At 3 years, OS was 100% and 48.4% (95% CI, 18.7%-73.0%) for the IC and control groups (*P* = .016), respectively (**Figure 2**). Two patients from the IC group experienced locoregional recurrence, and 2 patients from the control group experienced metastatic recurrence.

### Outcomes Based on CCI Score

**Figure 3.**
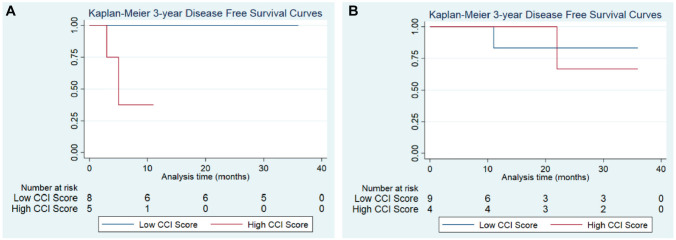
Disease-free survival by Charlson Comorbidity Index (CCI) scores. (A) For the induction chemotherapy group at 1 year, disease-free survival was observed at 83.3% for patients with low CCI scores and 100% for high CCI scores (*P* = .414). (B) For the control group, disease-free survival was as follows (by low vs high scores, respectively): 1 year, 100% vs 37.5% (*P* = .022); 2 years, 100% vs 37.5% (*P* = .022); and 3 years, 100% vs 37.5 (*P* = .023).

Using CCI, we conducted subanalyses based on comorbidities calculated for each patient. In a global comparison of high vs low CCI scores, patients with higher CCI scores demonstrated worse OS outcomes at all follow-up intervals, independent of treatment group (Supplemental Figures S1-S5, available online). Similarly, DFS rates were significantly reduced for control patients with high vs low CCI scores at 1 year (37.5% vs 100%; 95% CI, 1.1%-80.8%; *P* = .022; **Figure 3A**). In this subanalysis, OS outcomes for the IC group could not be calculated due the 100% survival rate at 3 years following treatment completion. Within the IC group, DFS rates were compared between patients with low and high CCI scores. Of note, patients receiving IC maintained comparable DFS rates at each interval, regardless of CCI score (**Figure 3B**).

## Discussion

A growing body of literature suggests a role of induction therapy in SNSCC. Benefits include organ preservation, increasing surgical outcomes, and potential for improved OS, particularly in patients who experienced a favorable tumor response.^19-22^ Our findings in this single-institutional analysis indicate that induction therapy prior to definitive management of locally advanced SNSCC improved survival outcomes as compared with SOC. Induction therapy was well tolerated and safe and resulted in high responses rates (84.6%) with either a complete or partial response. Our high OS rates are similar to what has been observed in SNSCC studies in patients experiencing partial response or better to IC. Hanna et al reported 77% 2-year OS, and Ock et al reported 84.6% 3-year OS (**Table 2**).^17,19^

**Table 2.** Literature on Overall Survival Rates for Patients With SNSCC Treated With Induction Chemotherapy.

Study: induction regimen	No.	Overall survival, %
Abdelmeguid et al^31^		2 y, 61.4; 3 y, 51.7
Platinum, taxane	41	
Platinum, taxane, 5FU	34	
Platinum, taxane, ifosfamide	26	
Platinum, taxane, cetuximab	8	
Platinum, 5FU	10	
Platinum, ifosfamide	2	
Platinum, gemcitabine	1	
Platinum, etoposide	1	
Ock et al ^17^		3 y, 84.6 (PR), 25 (SD/PD)
Docetaxel, 5FU, cisplatin	11	
Docetaxel, 5FU	8	
Docetaxel, cisplatin	2	
Noronha et al^32^		2 y, 41; 3 y, 35
Docetaxel, cisplatin	10	
Docetaxel, carboplatin	5	
Paclitaxel, cisplatin	15	
Paclitaxel, carboplatin	4	
Docetaxel, cisplatin, 5FU	7	
Farrell et al^33^		2 y, 64.1
Not specified		
Hanna et al^19^		2 y, 67, 77 (PR)
Taxane, platinum	14	
Taxane, platinum, ifosfamide	14	
Taxane, platinum, 5FU	9	
Taxane, 5FU	9	

Abbreviations: 5FU, 5-fluorouracil; PD, progressive disease; PR, partial response; SD, stable disease; SNSCC, sinonasal squamous cell carcinoma.

In this study, we used the CCI to measure individual health based on a patient’s comorbidities. While not statistically different, the IC cohort had a higher mean CCI score than the SOC cohort. Nevertheless, 3-year OS remains statistically improved in this group. This indicates that the combination of carboplatin, paclitaxel, and cetuximab is a well-tolerated induction regimen even in patients with multiple comorbidities.

While uncommon, recurrence patterns were different between the IC and control groups. Patients treated with IC had no occurrences of distant metastatic disease, while 2 patients in the control group ultimately progressed with metastatic disease. A meta-analysis of head and neck squamous cell carcinoma showed that IC statistically reduced rates of distant metastasis but had no effect on locoregional control^28^; this finding was also observed in our study. We hypothesize that this differential pattern of recurrence may have influenced OS outcomes.

Important limitations of this study must be acknowledged. First, the study analysis was retrospective and comprised patients treated over a 20-year period. During this time, there has been a shift in treatment practices, including the use of endoscopic approaches rather than an open approach. Second, given the rarity of the SNSCC, the sample size was small, and we were unable to include enough patients treated with IC to adequately power our study. Nevertheless, we report one of the largest cohorts with detailed IC regimens available in the current literature. In the modern era, induction therapy consists of only multiagent chemotherapy and not concurrent chemoradiation therapy. The small number of IC cases prompted our decision to include the 3 patients treated with neoadjuvant cisplatin and radiotherapy. All patients were treated prior to 2010, when this strategy was more commonly accepted. Additionally, no patients received induction therapy with a TPF regimen (5-flourouracil, platinum, docetaxel). However, numerous studies have shown benefit from the Kies regimen IC in head and neck squamous cell carcinoma.^9-12^ At our institution, the preference for the Kies regimen is driven by decreased toxicities, improved tolerability, and comparable efficacy to TPF.^10^ Third, the CCI tool is limited in its predictive capabilities and simply confers a crude estimate of 10-year mortality related to comorbid disease. Fourth, selection bias is inherently present in this study design, although we present findings on a clinically homogeneous high-risk population with >90% having T4 disease. These patients are most appropriate for consideration of IC.

At our institution and a growing number of academic centers, all patients with locally advanced SNSCC are evaluated for IC. Our threshold for treatment with IC is high, based on results from our data and as well as others (**Table 2**). Primary reasons to forgo IC include patient preference or existing conditions such as severe peripheral neuropathy. While most patients are treated with surgery for their definitive therapy following induction therapy, the use of concurrent chemoradiation therapy in patients with a dramatic response to IC is gaining favor. In sinonasal undifferentiated carcinoma, patients with a response to IC received concurrent chemoradiation therapy while nonresponders received surgery.^19^ More data evaluating this topic are required and would benefit from a prospective clinical study.

## Conclusion

The addition of induction therapy in the treatment of locoregionally invasive SNSCC shows promising survival outcomes. Our findings suggest that patients with high-risk disease who are treated with IC experience better OS outcomes as compared with similarly matched patients treated with the current SOC. This finding was consistent even among patients who had a higher burden of comorbidities. In light of these findings, we look to the results of a randomized clinical trial comparing IC vs non-IC treatment in SNSCC.^29,30^

## Supplemental Material

sj-docx-1-oto-10.1177_01945998221083097 – Supplemental material for Sinonasal Squamous Cell Carcinoma Survival Outcomes Following Induction Chemotherapy vs Standard of Care Therapy Click here for additional data file. Supplemental material, sj-docx-1-oto-10.1177_01945998221083097 for Sinonasal Squamous Cell Carcinoma Survival Outcomes Following Induction Chemotherapy vs Standard of Care Therapy by Alexander T. Murr, Nicholas R. Lenze, Jared M. Weiss, Juneko E. Grilley-Olson, Shetal A. Patel, Colette Shen, Bhishamjit S. Chera, Adam M. Zanation, Brian D. Thorp and Siddharth H. Sheth in Otolaryngology–Head and Neck Surgery
