# Supplementary material for: Sinonasal Squamous Cell Carcinoma Survival Outcomes Following Induction Chemotherapy vs Standard of Care Therapy
Source: Otolaryngol Head Neck Surg. 2022 Mar 8;167(5):846–51. doi: 10.1177/01945998221083097 (PMC9630958; doi:10.1177/01945998221083097)
Supplement: sj-docx-1-oto-10.1177_01945998221083097 – Supplemental material for Sinonasal Squamous Cell Carcinoma Survival Outcomes Following Induction Chemotherapy vs Standard of Care Therapy [file sj-docx-1-oto-10.1177_01945998221083097.docx]

**SUPPLEMENTAL MATERIAL**

**Survival Outcomes Following Induction Chemotherapy Compared to Standard of Care Therapy for Sinonasal Squamous Cell Carcinoma**

Alexander T. Murr BS^1^; Nicholas R. Lenze MD MPH^1^; Jared M. Weiss, MD^2^; Juneko E. Grilley-Olson, MD^2^; Shetal A. Patel, MD, PhD^2^; Colette Shen, MD PhD^3^; Bhishamjit S. Chera, MD^3^; Adam M. Zanation, MD^1^; Brian D. Thorp^1^, MD; Siddharth H. Sheth, DO, MPH^2^

^1^Department of Otolaryngology—Head & Neck Surgery, The University of North Carolina at Chapel Hill, Chapel Hill, NC, USA

^2^Division of Medical Oncology, Department of Medicine, The University of North Carolina at Chapel Hill, Chapel Hill, NC, USA

^3^Department of Radiation Oncology, The University of North Carolina at Chapel Hill, Chapel Hill, NC, USA

**Supplemental Figure 1: Disease Free Survival differentiated by CCI Scores.** 3-year, Low 97.1% versus High 48.6% (p=0.041). 2-year, Low 97.1% versus High 48.6% (p=0.041). 1-year, Low 97.1% versus High 72.9%; (p=0.166).


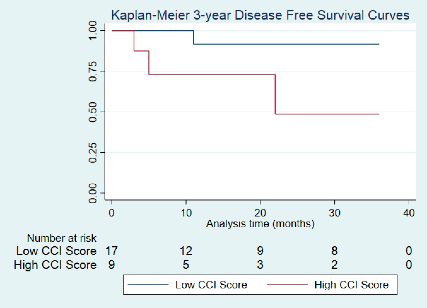


**Supplemental Figure 2: Overall Survival differentiated by CCI score.** 3-year Low Score 88.9% versus High 38.1% (p=0.004). 2-year, Low 100% versus High 50.8% (p=0.004). 1-year, Low 100% versus High 63.5% (p=0.013).


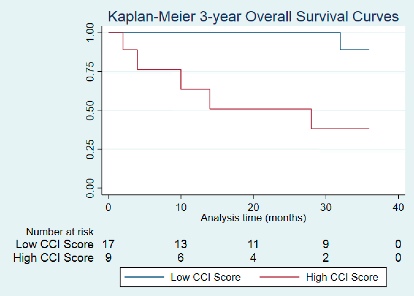


**Supplemental Figure 3: 3-year OS by high vs. low CCI score in Control Patients.** Low CCI Score 80.0% (95% CI 20.4% to 96.9%), High CCI Score 0%; p=0.001.


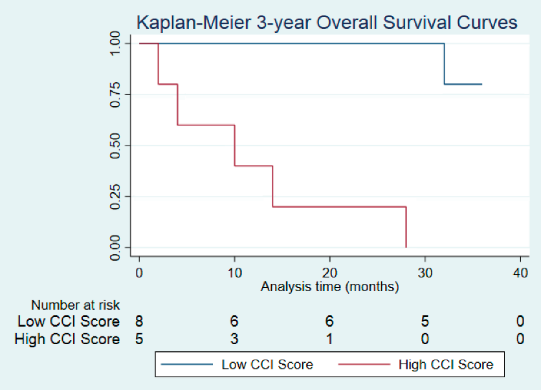


**Supplemental Figure 4: 3-year DFS by high vs. low CCI score in Induction Patients.** Low CCI Score 83.3% (95% CI 27.3% to 97.5%), High CCI Score 66.7% (95% CI 5.4% to 94.5%); p=0.886.


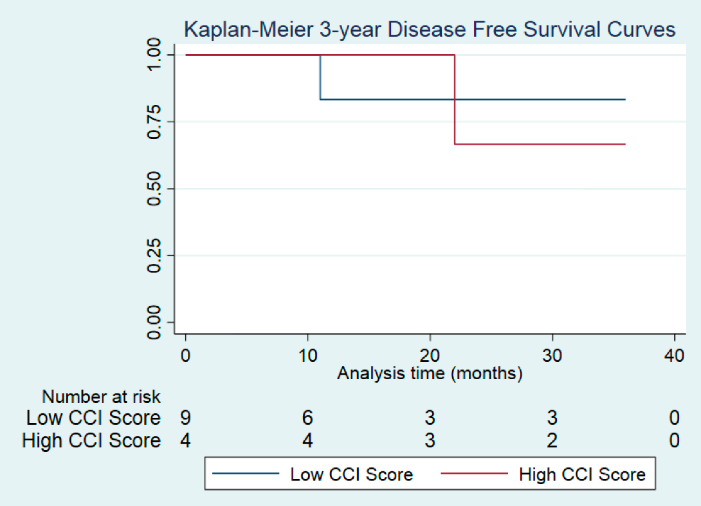


**Supplemental figure 5: 3-year DFS by high vs. low CCI score in Control Patients.**

Low CCI Score 100%, High CCI Score 37.5% (95% CI 1.1% to 80.8%); p=0.023.


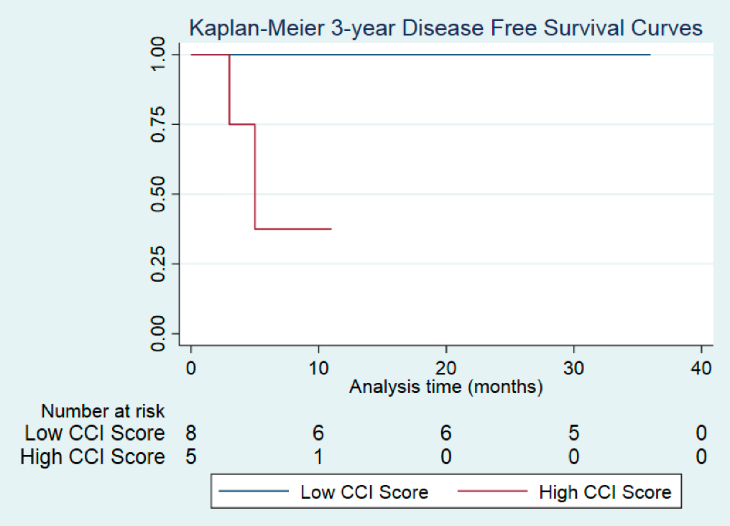


**Supplemental Table 1: Induction chemotherapy group characteristics**

| **Tumor Type** | **Tumor Location** | **Tumor Stage** | **Induction Therapy**  **Indication** | **Induction Therapy** | **Best overall response to IC** | **Grade >3 Treatment Related Toxicity to IC** | **Definitive Therapy** | **Surgical Margins** | **Adjuvant Chemotherapy** | **Adjuvant**  **Radiation Therapy** | **Recurrence**  **Following Treatment Completion** |
| --- | --- | --- | --- | --- | --- | --- | --- | --- | --- | --- | --- |
| Primary | Maxillary Sinus | T4aN0M0 | Improved CRT outcomes | carboplatin  paclitaxel  cetuximab | PR | None | Cisplatin 40mg/m^2^ x 4 cycles, radiation to 74 Gy | n/a | None | None | None |
| Primary | Maxillary Sinus | T4aN0M0 | Organ preservation | carboplatin  cetuximab  abraxane | SD | Type 1 HSR | Surgery | Pos | OSH | OSH | None |
| Recurrent | Maxillary Sinus | T4aN0M0 | Improved surgical resection | carboplatin  paclitaxel  cetuximab | PR | None | Cisplatin 40mg/m^2^ x 6 cycles, radiation to 71 Gy | n/a | None | None | None |
| Primary | Maxillary Sinus | T4aN0M0 | Organ preservation | carboplatin  paclitaxel | PD | None | Surgery | Pos | OSH | OSH | None |
| Primary | Maxillary Sinus | T4bN0M0 | Improved surgical resection | carboplatin  paclitaxel | PR | None | Surgery | Neg | Cisplatin 100mg/m^2^ x 3 cycles | 63 Gy | None |
| Primary | Ethmoid Sinus | T4bN0M0 | Organ preservation | Cisplatin  RT (75 Gy) | PR | None | Surgery | Neg | None | None | None |
| Primary | Maxillary Sinus | T4aN0M0 | Organ preservation | carboplatin  paclitaxel  cetuximab | PR | None | Surgery | Neg | Cisplatin 20 mg/m^2^ x 6 cycles | 74 Gy | None |
| Primary | Maxillary Sinus | T4aN0M0 | Organ preservation | carboplatin  paclitaxel  cetuximab | PR | None | Surgery | Neg | None | OSH | None |
| Primary | Maxillary Sinus | T4bN2cM0 | Improved surgical resection | Cisplatin  RT (70 Gy) | PR | None | Surgery | Neg | None | none | None |
| Primary | Maxillary Sinus | T4aN0M0 | Improved surgical resection | carboplatin  paclitaxel  cetuximab | PR | Neutropenia | Surgery | Pos | None | 60 Gy | None |
| Primary | Maxillary Sinus | T3N0M0 | Organ preservation | carboplatin  Paclitaxel  cetuximab | PR | None | Surgery | Neg | None | 60 Gy | None |
| Primary | Maxillary Sinus | T4aN0M0 | Organ preservation | carboplatin  paclitaxel  cetuximab | PR | None | Surgery | Neg | OSH | OSH | locoregional |
| Primary | Nasal  Cavity | T4aN0M0 | Organ preservation | Cisplatin  RT (60Gy) | CR | Nephrotoxicity | Surgery | Pos* | None | None | locoregional |

OSH = Therapy performed at outside hospital

*Unable to confirm margin status

**Supplemental Table 2: Control group characteristics**

| **Tumor Type** | **Original Tumor Location** | **Tumor Stage** | **Definitive Therapy** | **Surgical Margins** | **Adjuvant Chemotherapy** | **Adjuvant Radiation**  **Therapy** | **Recurrence** |
| --- | --- | --- | --- | --- | --- | --- | --- |
| Primary | Maxillary sinus | T4aN0M0 | Surgery | Pos | Cisplatin 100mg/m^2^ x  3 cycles | 70 Gy | Distant |
| Primary | Maxillary sinus | T4aN0M0 | Surgery | Neg | Weekly  carbo/taxol x  8 cycles | 70 Gy | Distant |
| Primary | Nasal cavity | T4aN0M0 | Surgery | Neg | None | 65 Gy | None |
| Primary | Nasal cavity | T4aN0M0 | Surgery | Neg | None | OSH | None |
| Primary | Maxillary sinus | T4N2bM0 | Surgery | Neg | Cisplatin 30mg/m^2^ x  6 cycles | 70 Gy | None |
| Primary | Nasal cavity | T4aN0M0 | Surgery | Neg | None | 58 Gy | None |
| Primary | Nasal cavity | T4bN0M0 | Surgery | Neg | None | 59 Gy | None |
| Primary | Ethmoid sinus | T4aN0M0 | Surgery | Neg | Cisplatin  30mg/m^2^ x  6 cycles | 60 Gy | None |
| Recurrent | Ethmoid sinus | T4aN0M0 | Surgery +  Intraop Rads | Neg | None | None | None |
| Primary | Maxillary sinus | T4bN0M0 | CRT | n/a | None | None | Persistent disease; salvage surgery |
| Primary | Clivus | T4N0M0 | CRT | n/a | None | None | None |
| Primary | Maxillary sinus | T4N0M0 | CRT | n/a | None | None | None |
| Primary | Septum | T4aN0M0 | Surgery | Pos | Cisplatin  40mg/ m^2^ x  6 cycles | 60 Gy | None |

CRT = chemoradiation therapy, OSH = Therapy performed at outside hospital
